# Analysis of Emission Effects Related to Drivers’ Compliance Rates for Cooperative Vehicle-Infrastructure System at Signalized Intersections

**DOI:** 10.3390/ijerph15010122

**Published:** 2018-01-12

**Authors:** Ruohua Liao, Xumei Chen, Lei Yu, Xiaofei Sun

**Affiliations:** 1 MOE Key Laboratory for Urban Transportation Complex Systems Theory and Technology, School of Traffic and Transportation, Beijing Jiaotong University, Beijing 100044, China; 11251037@bjtu.edu.cn; 2 College of Science, Engineering and Technology, Texas Southern University, Houston, TX 77004, USA; yu_lx@tsu.edu; 3 Beijing Capital International Airport Co., Ltd., Beijing 100621, China; sunxiaofei910@163.com

**Keywords:** signalized intersection, cooperative vehicle-infrastructure system, emissions, drivers’ compliance rates

## Abstract

Unknown remaining time of signal phase at a signalized intersection generally results in extra accelerations and decelerations that increase variations of operating conditions and thus emissions. A cooperative vehicle-infrastructure system can reduce unnecessary speed changes by establishing communications between vehicles and the signal infrastructure. However, the environmental benefits largely depend on drivers’ compliance behaviors. To quantify the effects of drivers’ compliance rates on emissions, this study applied VISSIM 5.20 (Planung Transport Verkehr AG, Karlsruhe, Germany) to develop a simulation model for a signalized intersection, in which light duty vehicles were equipped with a cooperative vehicle-infrastructure system. A vehicle-specific power (VSP)-based model was used to estimate emissions. Based on simulation data, the effects of different compliance rates on VSP distributions, emission factors, and total emissions were analyzed. The results show the higher compliance rate decreases the proportion of VSP bin = 0, which means that the frequencies of braking and idling were lower and light duty vehicles ran more smoothly at the intersection if more light duty vehicles complied with the cooperative vehicle-infrastructure system, and emission factors for light duty vehicles decreased significantly as the compliance rate increased. The case study shows higher total emission reductions were observed with higher compliance rate for all of CO_2_, NO_x_, HC, and CO emissions. CO_2_ was reduced most significantly, decreased by 16% and 22% with compliance rates of 0.3 and 0.7, respectively.

## 1. Introduction

At signalized intersections, traffic flows approach from different directions. The unknown remaining time of the signal phase generally results in extra accelerations and decelerations. These unnecessary accelerations and decelerations cause longer travel times, more complex driving modes, and increased emissions. To optimize the traffic flow and reduce harmful vehicle emissions, various cooperative vehicle-infrastructure systems (CVIS) have been developed in numerous countries. The cooperative vehicle-infrastructure system is an intelligent system that can collect the information provided by traffic infrastructure and vehicles, such as signal phases, and the vehicle’s real-time speed and position [1]. Using the system, it is feasible to advise speed for each vehicle. So, the cooperative vehicle-infrastructure system is recognized to have the potential to improve traffic safety and mobility. In the United States, the strategies of Vehicle Infrastructure Integration [2] and Cooperative Vehicle-Highway Automation Systems [3] have been developed in recent years. Smartway, based on a cooperative vehicle-infrastructure system, was also implemented in Japan [4]. At signalized intersections, a cooperative vehicle-infrastructure system can improve efficiency by providing guidance information, such as the signal phase and speed advice, through communication between vehicles and the signal infrastructure. If a driver accepts the real-time guidance information, he or she can accelerates, decelerates, or maintains the vehicle’s original speed, thus enabling the vehicle to stop slowly or run smoothly through a signalized intersection. The potential environmental benefits of this technology are also significant. In China, the benefits are enormously appealing when the trend for energy conservation and emission reduction becomes irreversible nationwide. Nevertheless, drivers with different personal attributes may have different attitudes to traffic guidance at a signalized intersection when they are under different external conditions. When drivers were asked in a stated preference survey, some drivers stated that they preferred not to accept the guidance information, and they trusted themselves more than they trusted a new technology [5]. Therefore, the benefits largely depend on drivers’ compliance with the cooperative vehicle-infrastructure system. To evaluate the effects of drivers’ compliance behaviors on emissions, this study developed a simulation model for a signalized intersection by using VISSIM. Simulated light duty vehicles were integrated into the cooperative vehicle-infrastructure system in the simulation model. The compliance rate is defined as the proportion of the number of vehicles that accept guidance information to the total number of vehicles at a signalized intersection. The emissions under different compliance rates were estimated using a vehicle specific power (VSP)-based modeling approach. The results of this study can not only promote the implementation of cooperative vehicle-infrastructure system, an innovative transport management technology which enables communication between vehicles and traffic infrastructure in smart cities and communities, but can also facilitate developing strategies to reduce the environmental impact of cities and ensure environmental sustainability.

## 2. Literature Review

In recent years, researchers have studied the environmental benefits of cooperative vehicle-infrastructure systems at signalized intersections. Bhavsar et al. studied the energy consumption reduction of plug-in hybrid electric vehicles (PHEVs) equipped with connected vehicle technology (CVT). The results indicated that the energy savings of CVT-supported PHEVs can increase by 31% to 35% when the PHEVs receive signal timing and headway data [6]. Most studies about the emission effects of cooperative vehicle-infrastructure systems have been conducted on the basis of simulation technology. Englund et al. proposed a wave-based control mechanism for a cooperative vehicle-infrastructure system at a highly utilized intersection. The application contributed to the overall efficiency of all traffic, including both cooperative vehicles and non-cooperative vehicles. The emissions of carbon dioxide decreased, and the travel speeds increased [7]. Ubiergo and Jin proposed a simulator integrated with a vehicle-infrastructure system for simulating the behaviors of vehicles at signalized intersections and for calculating travel delays in queues, vehicle emissions, and fuel consumption levels; they presented a hierarchical green driving strategy that is based on feedback control for smoothing stop-and-go traffic in signalized networks. Simulation results demonstrated savings of approximately 15% in travel delays and approximately 8% in greenhouse gas emissions [8]. Kamalanathsharma and Rakha developed an eco-cooperative adaptive cruise control model that used infrastructure-to-vehicle communication to predict future constraints on a vehicle’s trajectory and optimize its trajectory to minimize the vehicle’s fuel consumption. The model was demonstrated to provide fuel savings within the vicinity of signalized intersections in the range of 5% to 30% in the United States [9]. Ni et al. introduced an adaptive traffic light control algorithm for intersections based on vehicle-to-vehicle and vehicle-infrastructure system communication technologies; the algorithm can dynamically adjust the timing and phasing of a traffic light and recommend speeds for drivers. Simulation results indicated that the scheme exhibited higher performance in reducing average CO_2_ emissions compared with a fixed-time control scheme [10]. Li et al. and Wu et al. have proposed an advanced driving alert system, which can provide traffic signal information to drivers in real time. Simulation results showed that this system could be applied to avoid significant amounts of energy consumption and emissions caused by unnecessary accelerations and hard braking at signalized intersections [11,12].

Some studies aimed at the impacts of market penetration rates of controlled vehicles. Yang et al. proposed an eco-cooperative adaptive cruise control algorithm that can compute the fuel-optimum trajectory through a signalized intersection. Simulation analyses demonstrated that for single-lane approaches, the higher the market penetration rate was, the larger fuel consumption that was saved [13]. Katsaros et al. developed the Green Light Optimized Speed Advisory (GLOSA) application and monitored the impacts of GLOSA on fuel. The simulations indicated that higher penetration rates result in more benefits in fuel saving. The environmental effect started to be more visible after the rate was higher than 50% [14].

In summary, numerous studies have shown that implementing cooperative vehicle-infrastructure systems can reduce emissions and save fuel at signalized intersections. However, notably, the effectiveness of a cooperative vehicle-infrastructure system is directly affected by drivers’ compliance behaviors with the guidance information; this implies that the environmental benefits associated with the system largely depend on the drivers’ rates of compliance with the guidance information. So far, a very limited number of studies have analyzed relationships between emissions and driver compliance rates. Even some studies considered the impact from market penetration rates of vehicles controlled by cooperative vehicle-infrastructuresystems; they assumed that the driver compliance rate is 1. However, there is a difference between applying market penetration rate and drivers’ compliance rate. Market penetration rate is influenced by the development level of intelligent transportation and traffic policies. Market penetration rate cannot reflect the drivers’ different attitudes to traffic guidance when they are in different external conditions. In this context, this study strived to quantify the emissions under different driver compliance rates by using a VSP-based emission modeling approach that entailed simulating a signalized intersection with a cooperative vehicle-infrastructure system.

## 3. Methodology

**Figure 1:**
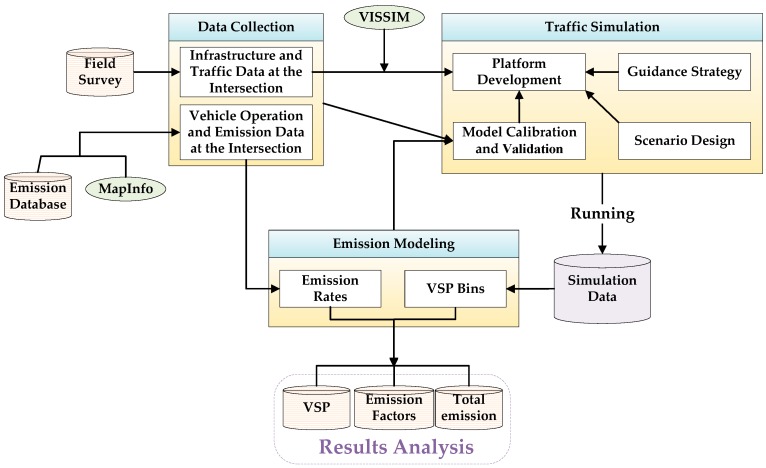
Framework of study approach.

Figure 1 presents the framework of the study approach, which includes data collection, traffic simulation, emission modeling, and results analysis.

First, in the data collection, an infrastructure data set and a traffic data set were collected at a real-world intersection through a field survey. These data were used to establish the simulation platform. A vehicle operations data set and an emission data set were selected using the MapInfo from an existing vehicle emission database for calculating emission rates.

Second, in the traffic simulation, a simulation platform was developed using VISSIM. The model was calibrated and validated using the collected data. Moreover, the model was validated by comparing the observed and simulated VSP distributions. The VSP distributions were calculated using the emission model. Subsequently, a cooperative vehicle-infrastructure control strategy was implemented using the COM external program interface in VISSIM. With the control strategy, the guidance information could be provided to vehicles. Scenarios were designed and simulated. After running the traffic simulation, the simulated data under different compliance rates were obtained.

Third, the emission model was developed. Based on the simulation data and the emission rates, the VSP distributions, emission factors, and total emissions were estimated for different compliance rates.

Finally, a comparative analysis was conducted based on the calculation results to quantify the effects of drivers’ compliance rates on emissions at the signalized intersection equipped with the cooperative vehicle-infrastructure system.

### 3.1. Data Collection

The collected data include infrastructure data, traffic data, vehicle operation data, and emission data. These data were used for the simulation platform development, model calibration, model verification, and emission rates calculation.

#### 3.1.1. Infrastructure and Traffic Data

**Figure 2:**
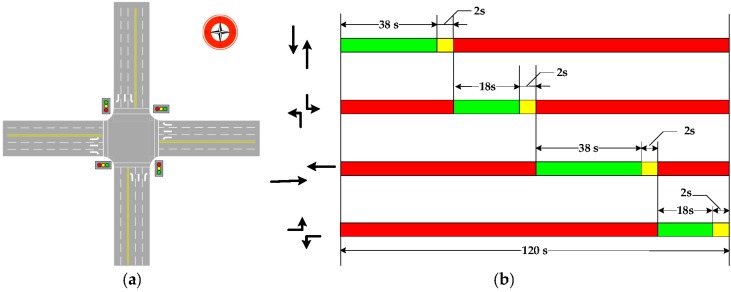
Study intersection: (**a**) intersection layout; (**b**) signal phases.

A signalized intersection of Fuchengmen Avenue and Xisi Avenue in Beijing was selected as the test bed to develop a simulation model in this research. At this intersection, geometry, signal, and traffic flow data have been collected. Geometry data include the size of the intersection and the lanes of each approaching direction. As shown in Figure 2a, each approaching direction comprises three lanes, which are a left-turn lane, through lane, and right-turn lane. The existing signal phases have been investigated. Through the field survey, there was four-phase at the signalized intersection. In the south-north direction and the east-west direction, the durations of straight and left-turn green light phase were 38 s and 18 s, respectively. The durations of amber signal light were 2 s. The cycle length is 120 s. The actual signal phases were all used to develop the simulation platform. Figure 2b presents the existing signal phases. Traffic data include the type of vehicles and traffic volumes. These data were collected through a field survey. Some data were collected using camera, such as the existing signal phases.

Two types of vehicles run through the intersection: light duty vehicles and buses. Light duty vehicles are defined as cars with the gross vehicle weight ratings less than 8500 lbs. Light duty vehicles were selected for implementing the cooperative vehicle-infrastructure control. The vehicles randomly arrive at the signalized intersection. There is no platooning of vehicles with coordinated signals. The capabilities behind harmonic speed profiling with connected vehicle systems are enough.

#### 3.1.2. Vehicle Operating and Emission Data

Vehicle operating data, including second-by-second speed, acceleration, and geographic information, and emission data at the study intersection, were obtained from similar intersections in the vehicle emission database established by the Transportation Environmental Lab of Beijing Jiaotong University. The influence area of the signal at the intersection was defined as a circle with a radius of 150 m. The vehicle operating data in this area were selected using the buffer function of MapInfo [15].

Finally, a total of 40,000 second-by-second records of the vehicles’ operating and emission data at the intersection were collected, comprising 30,000 records of light duty vehicles and 10,000 records of buses. The vehicle operating and emission data were used to validate simulation model and estimate average emission rates for each VSP bin.

### 3.2. Traffic Simulation Considering Cooperative Vehicle-Infrastructure Control

To estimate emissions under different compliance rates, a simulation model of the intersection was developed using VISSIM. The cooperative vehicle-infrastructure system was developed and embedded in the model. The external program interface enabled adjusting drivers’ compliance rates.

#### 3.2.1. Platform Development

On the basis of the field data, this study developed a simulation model by using VISSIM [16]. The cooperative vehicle-infrastructure control was developed with Microsoft Visual Basic and embedded into the simulation model through COM [17]. The functions of the simulation model are outlined as follows:

- Providing signal status in real time;
- Collecting instantaneous vehicle operating data such as speed;
- Providing speed advice to drivers on the basis of the signal status and vehicle operating conditions.

#### 3.2.2. Model Calibration and Validation

**Figure 3:**
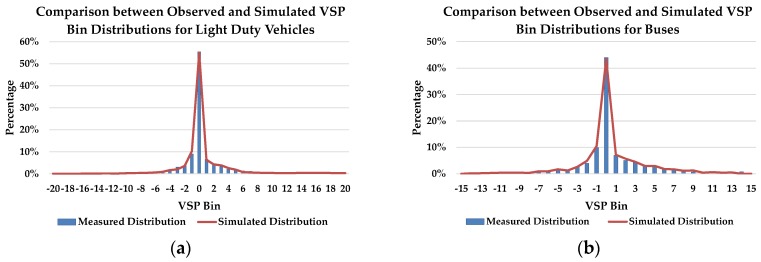
Comparison between observed and simulated VSP bin distributions: (**a**) light duty vehicles; (**b**) buses.

The simulation model was calibrated using an approach based on genetic algorithms to find optimal parameter combination so that the model could accurately present the actual running states of the vehicles. Driving behavior parameters in VISSIM that are sensitive to the traffic volume and speed are calibrated, such as maximum look ahead distance, the number of observed vehicles, average standstill distance, additive part of safety distance, and waiting time before diffusion. Each parameter was divided into four levels. After using the Genetic Algorithm, the optimal parameter combination could be found. [18]. After the calibration, the simulation model was further validated by comparing the observed data obtained from similar intersections in the vehicle emission database and simulated output files, to evaluate its reliability and to ensure that it could precisely estimate the emissions at the signalized intersection. Ten simulation runs were conducted with ten random number seeds. Observed and simulated VSP distributions are compared in Figure 3, indicating little difference between the observed and simulated VSP distributions. CO_2_, NO_x_, HC, and CO emissions were estimated using observed and simulated data. All of the mean absolute percentage errors were found to be within 10%, signifying that the simulation model was suitable for estimating the emissions.

#### 3.2.3. Guidance Strategy

**Figure 4:**
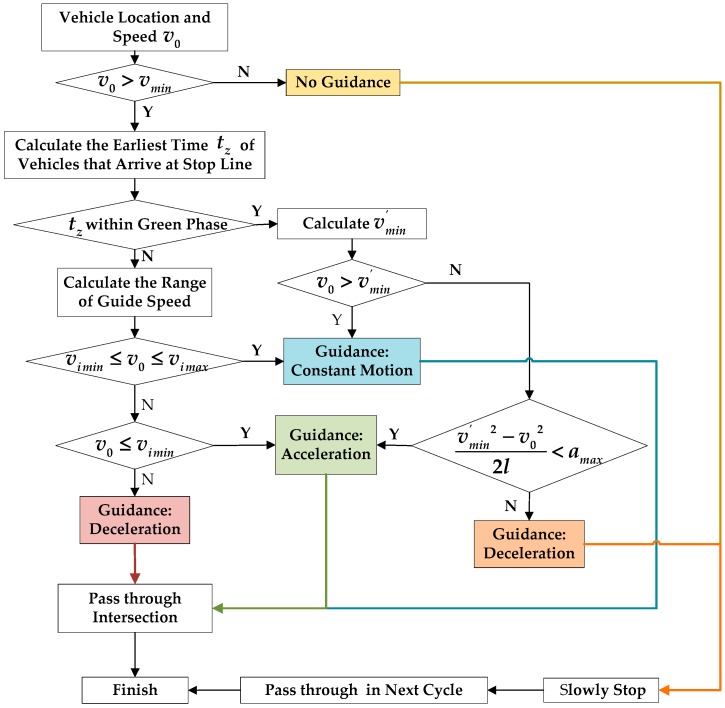
Guidance process.

The COM interface in VISSIM was used to implement the algorithm of the cooperative vehicle-infrastructure control. In this algorithm, drivers are guided with speed advice at the intersection. Restricted by regular routes and stations, buses are not provided with speed advice. Therefore, the cooperative vehicle-infrastructure system works only for light duty vehicles. When light duty vehicles enter the influencing area (a circle of radius 150 m), their instantaneous locations and speeds are sent to the control center. According to the real-time signal status, the control center determines the guidance speed and provides advice to the vehicles [12]. The guidance advice was proposed to make the vehicle pass through the intersection in the present green light phase or the next green light phase as much as possible. The comparison of the real-time speed of vehicle and a series of threshold velocities was conducted to determine the guidance strategies. Figure 4 presents the guidance process.

It should be noted that:

- To simplify the model, the vehicle waiting time is assumed to be less than one signal cycle length at the intersection;
- If the effect of the leading vehicle is considered, the earliest time at which vehicle A can arrive at the stop line should be calculated using Equation (1)

(1) $$t_{A}=t_{B}+t_{min}$$

where $t_{A}$ is the earliest time at which vehicle *A* can arrive at the stop line, $t_{B}$ is the actual time at which leading vehicle *B* arrives at the stop line, and $t_{min}$ is the minimum headway (2 s).

#### 3.2.4. Scenario Design

A total of 11 guidance compliance rates were entered into the developed simulation model to study their effects on emissions. The minimum compliance rate is 0, which indicates that no vehicle accepts the guidance information. The maximum compliance rate is 1, which indicates that all light duty vehicles accept the guidance information. The compliance rates were set at intervals of 0.1. The market penetration rates is 1. Considering the simulation randomness and sample size, the simulation was run five times with a unique random number seed for each run under each compliance rate. Each run of the simulation simulated 1200 s of events. The output data included the vehicle type, vehicle number, speed, acceleration, and simulation time. Based on the simulation outputs, the VSP bins and vehicle emissions were estimated and analyzed.

### 3.3. Emission Modeling

A VSP-based approach is employed to estimate emissions. The steps for emissions estimations are as follows.

#### 3.3.1. Division of VSP bins

As one of the parameters closely related to vehicle operating conditions, the VSP indicates the ratio of the vehicle’s output power over its mass. The VSP establishes an informative relationship between traffic emissions and driving activities, so the VSP-based model is a current trend of the emission models. MOVES, one of the VSP-based models, is a novel emissions model developed by EPA which can be used to calculate the emission factors of vehicles accurately. Based on the instantaneous speed and acceleration obtained from simulation, the VSP values for light duty vehicles and buses were calculated using Equation (2) [19]:

(2) $$\mathrm{VSP}=(Av+Bv^{2}+Cv^{3}+mva)/m$$

where *v* is the instantaneous speed in m/s, and *a* is the acceleration in m/s^2^. For light duty vehicles, *A* = 0.156461, *B* = 0.002002, *C* = 0.000493, and *m* = 1.4788. For buses, *A* = 0.746718, *B* = 0, *C* = 0.002176, and *m*= 9.06989.

VSP values were divided into VSP bins using the hierarchical clustering method, as presented in Equation (3). In this equation, the VSP data are arranged in 1 kW/ton intervals [20]:

(3) VSPbin=n,∀:VSP∈[n−0.5,n+0.5]

where, *n* is the integer from −20 to 20 for light duty vehicles and from −15 to 15 for buses.

#### 3.3.2. Calculation of Emission Rates

**Figure 5:**
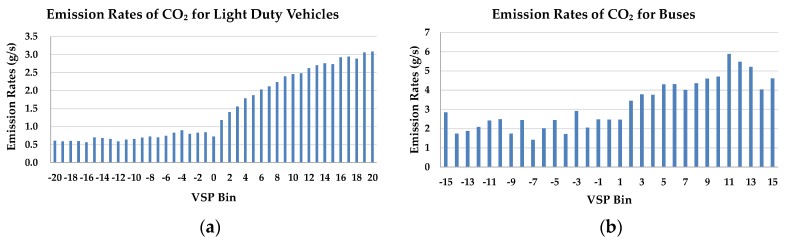
Emission rates of CO_2_ in each VSP bin: (**a**) Light duty vehicles; (**b**) Buses.

The average emission rates for each VSP bin were estimated using the vehicle operating data and emission data obtained from similar intersections in the vehicle emission database. The emission rates of CO, HC, NO_x_, and CO_2_ for light duty vehicles and buses were obtained. Examples of emission rates of CO_2_ in each VSP bin for light duty vehicles and buses are illustrated in Figure 5.

#### 3.3.3. Emission Estimations

Emission factors, which are defined as the amounts of emissions produced per kilometer, were calculated using Equation (4) [21].

(4) $$EF_{i}=\sum_{j}ER_{i,j}\times D_{j}\times T/L\times 1000$$

where *i* is the type of emission, $EF_{i}$ is the emission factor for emission type *i* (g/km), *j* is the VSP bin, $ER_{i,j}$ is the emission rate in the VSP bin *j* for emission type *i* (g/s), $D_{j}$ is the frequency of the VSP bin *j*, *T* is the total travel time (s), and *L* is the total travel distance (km).

Based on the quantities of light duty vehicles and buses, the total emissions were then estimated using Equation (5):

(5) $$Q_{i}=EF_{c,i}\times q_{c}\times L+EF_{b,i}\times q_{b}\times L$$

where $Q_{i}$ is the total emissions (kg) for emission type *i* in an approaching direction of the intersection (kg); $EF_{c,i}$ and $EF_{b,i}$ are the emission factors for light duty vehicles and buses for emission type *i* (g/km), respectively; $q_{c}$ and $q_{b}$ are the volumes of light duty vehicles and buses in an approaching direction; and *L* is a travel distance equal to 300 m at the studied intersection.

## 4. Results and Discussion

Based on the simulation platform and the emission model, the VSP distributions, emission factors, and total emissions under different compliance rates were calculated using the instantaneous speed and acceleration obtained from simulation. A comparative analysis was conducted to quantitatively evaluate the effects of drivers’ compliance behaviors on emissions at the studied intersection.

### 4.1. VSP Distributions under Different Compliance Rates

**Figure 6:**
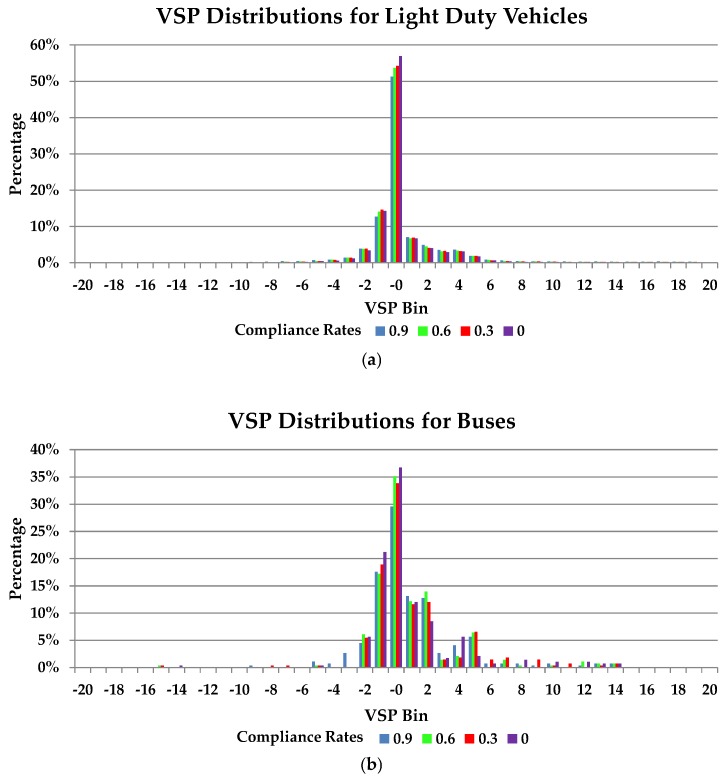
VSP distributions: (**a**) light duty vehicles; (**b**) buses.

VSP distributions of light duty vehicles and buses were calculated for 11 compliance rates ranging from 0 to 1 using Equations (2) and (3). The VSP distributions under compliance rates of 0, 0.3, 0.6, and 0.9 are illustrated in Figure 6 as examples.

Results showed that the VSP distributions of light duty vehicles were different for different compliance rates, as shown in Figure 6a. In particular, the difference reached its maximum in the VSP bin of 0. Calculations for low compliance rates had high proportions of entries in the VSP bin of 0. In the VSP bin of 0, the vehicle speed was 0 km/h. To a certain degree, the proportion of entries in the VSP bin of 0 represents the frequency of braking and idling (a vehicle is idling if its speed is 0 m/s and its acceleration is 0 m/s^2^). Therefore, it can be concluded that if the compliance rate is high, then the frequencies of braking and idling are low and light duty vehicles run more smoothly at the intersection.

Buses had no regular pattern in the VSP distributions under different compliance rates, as shown in Figure 6b. This is because buses were not controlled by the cooperative vehicle-infrastructure system at the intersection. The compliance rates of light duty vehicles have little influence on buses. 

### 4.2. Emission Factors under Different Compliance Rates

Emission factors under different compliance rates were estimated using Equation (4), and the results are shown in Table 1.

The results in Table 1 indicate that the emission factors of CO_2_, NO_x_, HC, and CO exhibited a downward trend for light duty vehicles with the increase of the compliance rate. Of the four emission types, the percentage of HC emission factor was reduced most significantly. However, the emission factors for buses did not show notable differences under different compliance rates of light duty vehicles. The fluctuation of emission factors for buses is within 3%.

**Figure 7:**
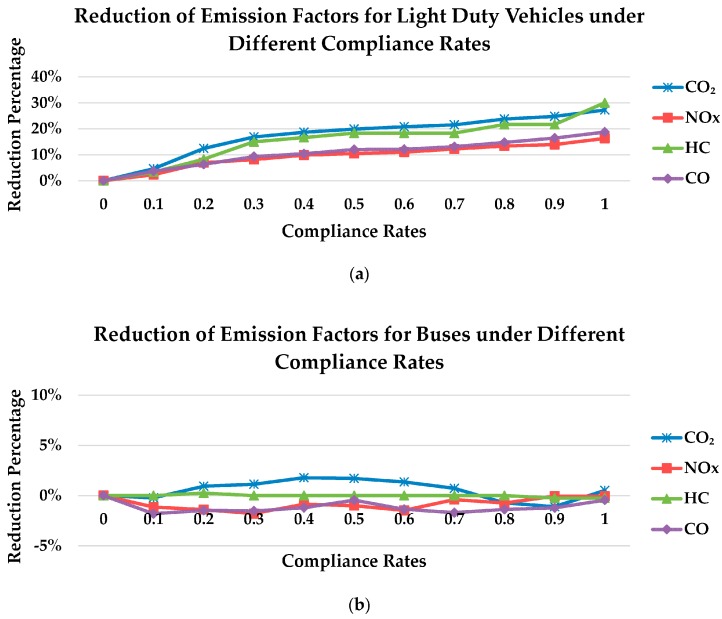
Reduction of emission factors under different compliance rates: (**a**) light duty vehicles; (**b**) buses.

In order to analyze the variation of emission factors more clearly, the reduction percentages of the emission factors under compliance rates ranging from 0.1 to 1 were estimated. The variations for light duty vehicles were quite significant and the reduction percentages of the emission factors for buses were low (Figure 7). Figure 7a shows higher compliance rate reduces emission factors for light duty vehicles for all emission types more significantly. This demonstrates that the cooperative vehicle-infrastructure system indeed generates environmental benefits. When the compliance rate was 1, the emission factors will reach their minimum value; the reduction percentages of CO_2_, NO_x_, HC, and CO were 27%, 16%, 30%, and 19%, respectively. Figure 7b shows no regular pattern and minor differences under different compliance rates. Bulleted lists look like this:

### 4.3. Total Emissions under Different Compliance Rates

**Figure 8:**
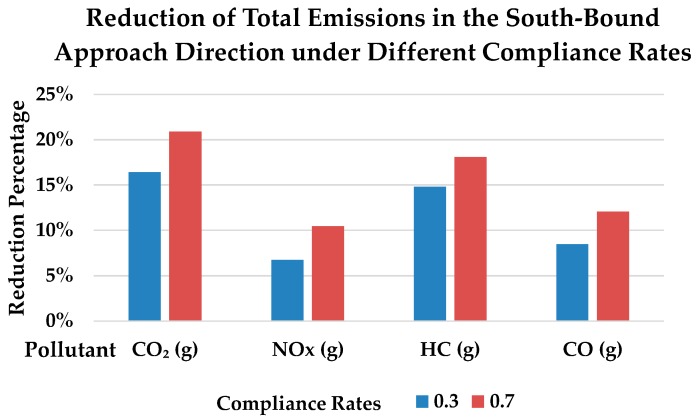
Reduction of total emissions in the southbound approach direction under different compliance rates.

To demonstrate the environmental benefits more intuitively, total emissions in the southbound approach of the intersection were estimated using Equation (5). Traffic volumes were collected at the signalized intersection of Fuchengmen Avenue and Xisi Avenue through the field survey. The volumes of light duty vehicles and buses through the approach were 429 pc/h (passenger car per hour) and 9 vehicles/h, respectively. The total vehicles emissions per hour were estimated for compliance rates of 0, 0.3, and 0.7 (Table 2). In addition, the reduction percentages of emissions under the compliance rates of 0.3 and 0.7, compared to compliance rate of 0, were calculated. Figure 8 shows higher total emission reduction were observed with higher compliance rate for all CO_2_, NO_x_, HC, and CO emissions. Among the four emission types, CO_2_ was reduced most significantly. Specifically, CO_2_ emissions were observed to decrease by 16% and 22% under compliance rates of 0.3 and 0.7, respectively.

## 5. Conclusions

This study investigated the effects of drivers’ compliance rates on emissions at a signalized intersection equipped with a cooperative vehicle-infrastructure system. A VISSIM-based simulation model of a signalized intersection was developed. Based on the simulation results under different compliance rates of light duty vehicles, the VSP distributions, emission factors, and total emissions were estimated. The effects of different compliance rates on emissions were analyzed by comparing the estimation results. The main findings are summarized as follows:

- A higher compliance rate decreases the proportion of VSP bin = 0, which means that the frequencies of braking and idling are lower, and light duty vehicles run more smoothly at the intersection if more light duty vehicles comply with the cooperative vehicle-infrastructure system.
- Emission factors of CO_2_, NO_x_, HC, and CO for light duty vehicles decreased significantly as the compliance rate increased. When the compliance rate was 1, the emission factors had minimums. The reduction percentages of CO_2_, NO_x_, HC, and CO were 27%, 16%, 30%, and 19%, respectively.
- Higher total emission reductions were obtained with higher compliance rate for all of CO_2_, NO_x_, HC, and CO emissions. CO_2_ was reduced most significantly, decreased by 16% and 21% with compliance rates of 0.3 and 0.7, respectively.

The findings of this study provide insights for transportation practitioners to implement emission reduction policies in cities and promote environmental sustainability. In the future, the analyses could be conducted at other types of signalized intersection using different signal phases or groups of intersections with cooperative vehicle-infrastructure systems. New studies on providing guidance strategies for buses and the vehicles platoons in cooperative vehicle-infrastructure systems will be conducted.

## Figures and Tables

**Table 1:** Emission factors of CO_2_, NO_x_, HC, and CO under different compliance rates.

Compliance Rate	Light Duty Vehicles (g/km)				Buses (g/km)			
	CO_2_	NO_x_	HC	CO	CO_2_	NO_x_	HC	CO
0.0	82.903	0.172	0.060	1.249	131.534	1.498	0.041	5.302
0.1	79.073	0.168	0.058	1.203	131.825	1.515	0.041	5.398
0.2	72.601	0.160	0.055	1.170	130.313	1.519	0.040	5.380
0.3	68.924	0.158	0.051	1.133	130.046	1.525	0.041	5.383
0.4	67.421	0.155	0.050	1.119	129.200	1.511	0.041	5.366
0.5	66.412	0.154	0.049	1.099	129.278	1.513	0.041	5.326
0.6	65.675	0.153	0.049	1.098	129.759	1.520	0.041	5.375
0.7	65.069	0.151	0.049	1.085	130.585	1.504	0.041	5.392
0.8	63.202	0.149	0.047	1.065	132.479	1.509	0.041	5.376
0.9	62.355	0.148	0.047	1.044	132.996	1.499	0.042	5.367
1.0	60.346	0.144	0.042	1.015	130.857	1.499	0.042	5.326

**Table 2:** Total emissions in the southbound approach direction under different compliance rates.

Compliance Rates	CO_2_ (g)	NO_x_ (g)	HC (g)	CO (g)
0	10,985.2977	25.7316	7.8204	173.4711
0.3	9182.6292	23.9946	6.6621	158.7363
0.7	8687.7843	23.0433	6.4047	152.5803

## Abbreviations

$v_{min}$	the minimum speed (20 km/h). Assume that if the vehicle speed is less than $v_{min}$, the vehicle is in the congestion. So the vehicle cannot be guided
$v_{max}$	the maximum speed (60 km/h)
$a_{max}$	the maximum acceleration (3 m/s^2^)
$v_{0}$	the real-time speed when the vehicle enters the influencing area
$t_{z}$	the earliest time at which the vehicle arrives at the stop line. If $t_{A}$ (the earliest time at which vehicle A can arrive at the stop line because of leading vehicle B) is later than $t_{z}$ in the guidance process, $t_{z}$ must be replaced by $t_{A}$
$v^{\prime }{}_{min}$	the minimum speed at which the vehicle can pass through the intersection in the present green light phase
$v_{imin}$	the minimum speed when the vehicle can pass through the intersection in the next green light phase
$v_{imax}$	the maximum speed when the vehicle can pass through the intersection in the next green light phase
*l*	the radius (150 m) of the influencing area
